# Supplementary material for: Sensitivity Analysis of Leakage Correction of GRACE Data in Southwest China Using A-Priori Model Simulations: Inter-Comparison of Spherical Harmonics, Mass Concentration and In Situ Observations
Source: Sensors (Basel). 2019 Jul 17;19(14):3149. doi: 10.3390/s19143149 (PMC6679240; doi:10.3390/s19143149)
Supplement: Supplementary file 1 [file sensors-19-03149-s001.pdf]

sensors

Article

Sensitivity Analysis of Leakage Correction of GRACE Data in Southwest China Using A-Priori Model Simulations: Inter-Comparison of Spherical Harmonics, Mass Concentration and In Situ Observations

Zhiyong Huang [1,2,3], Jiu Jimmy Jiao [1,2,3,*], Xin Luo [1,2,3], Yun Pan [4,5] and Chong Zhang [6,7]

[1] Department of Earth Sciences, The University of Hong Kong, Hong Kong 999077, China
[2] The University of Hong Kong-Shenzhen Research Institute (SRI), Shenzhen 518057, China
[3] The University of Hong Kong-Zhejiang Institute of Research and Innovation (HKU-ZIRI), Hangzhou 311305, China
[4] Beijing Laboratory of Water Resources Security, Capital Normal University, Beijing 100048, China
[5] State Key Laboratory Base of Urban Environmental Processes and Digital Modelling, Capital Normal University, Beijing 100048, China
[6] State Key Laboratory of Earth Surface Processes and Resource Ecology, Faculty of Geographical Science, Beijing Normal University, Beijing 100875, China
[7] Institute of Land Surface System and Sustainable Development, Faculty of Geographical Science, Beijing Normal University, Beijing 100875, China
* Correspondence: jjiao@hku.hk; Tel.: +852-2857-8246

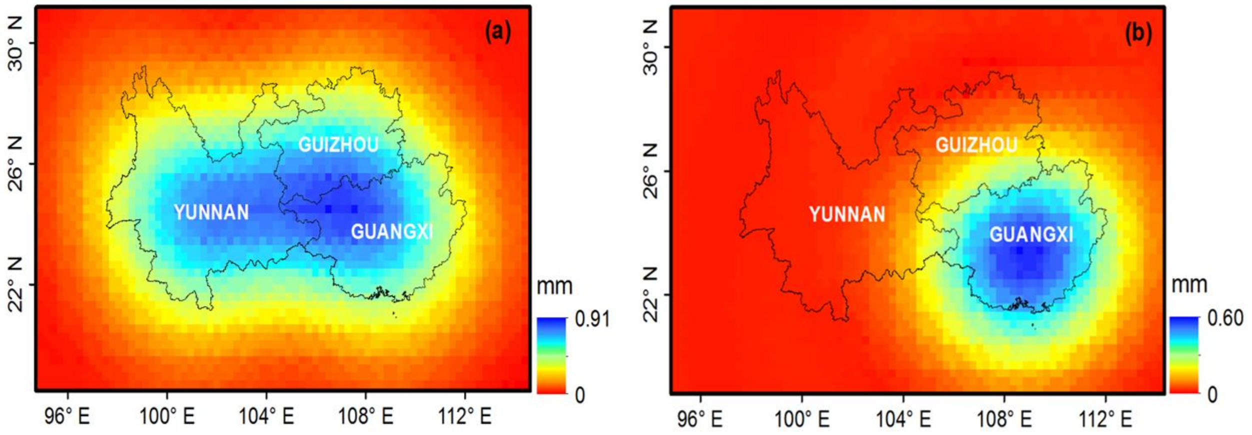

Figure S1. The filtered exact kernel functions used to constrain the regional averaged GRACE signals in (**a**) SW China and its subregion (**b**) Guangxi.

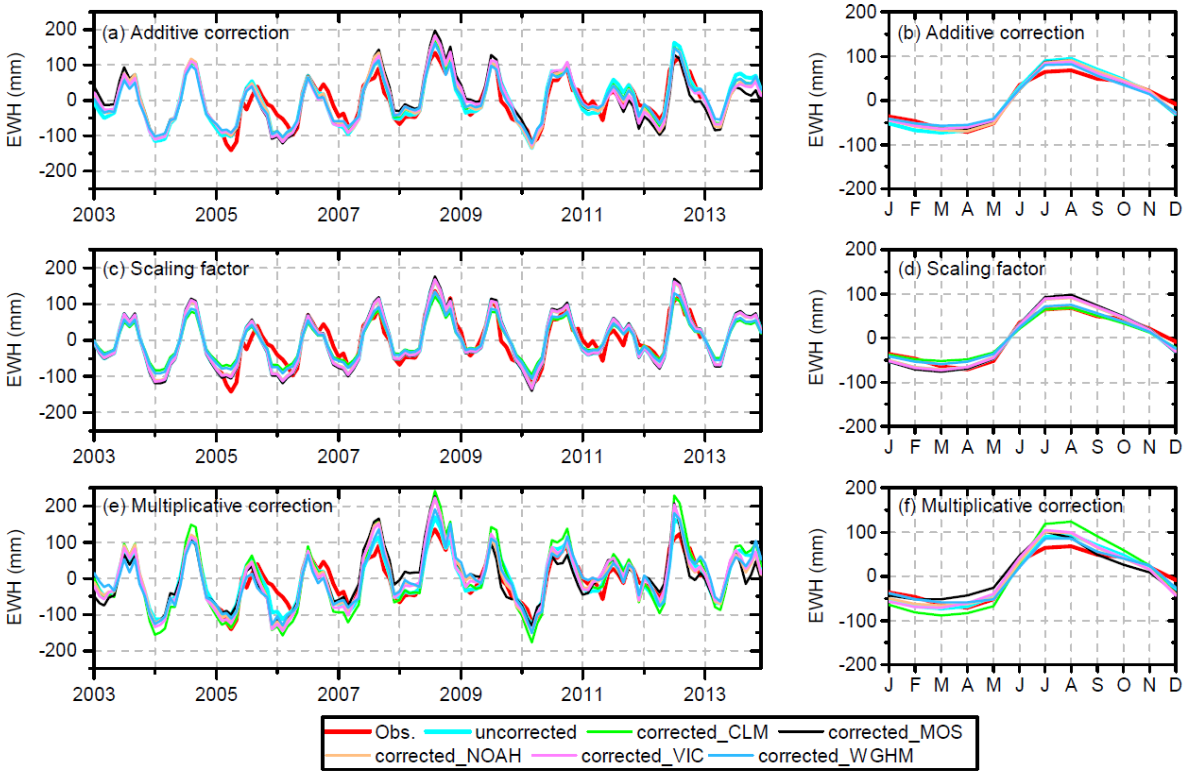

Figure S2. Left panel: Comparison of monthly time series of uncorrected and leakage corrected GRACE TWS with in situ observations of TWS for SW China. The leakage-corrected TWS is based on three different methods using the five model simulations as the a-priori information. Right panel: Multi-year mean annual cycle of the TWS in the left panel.

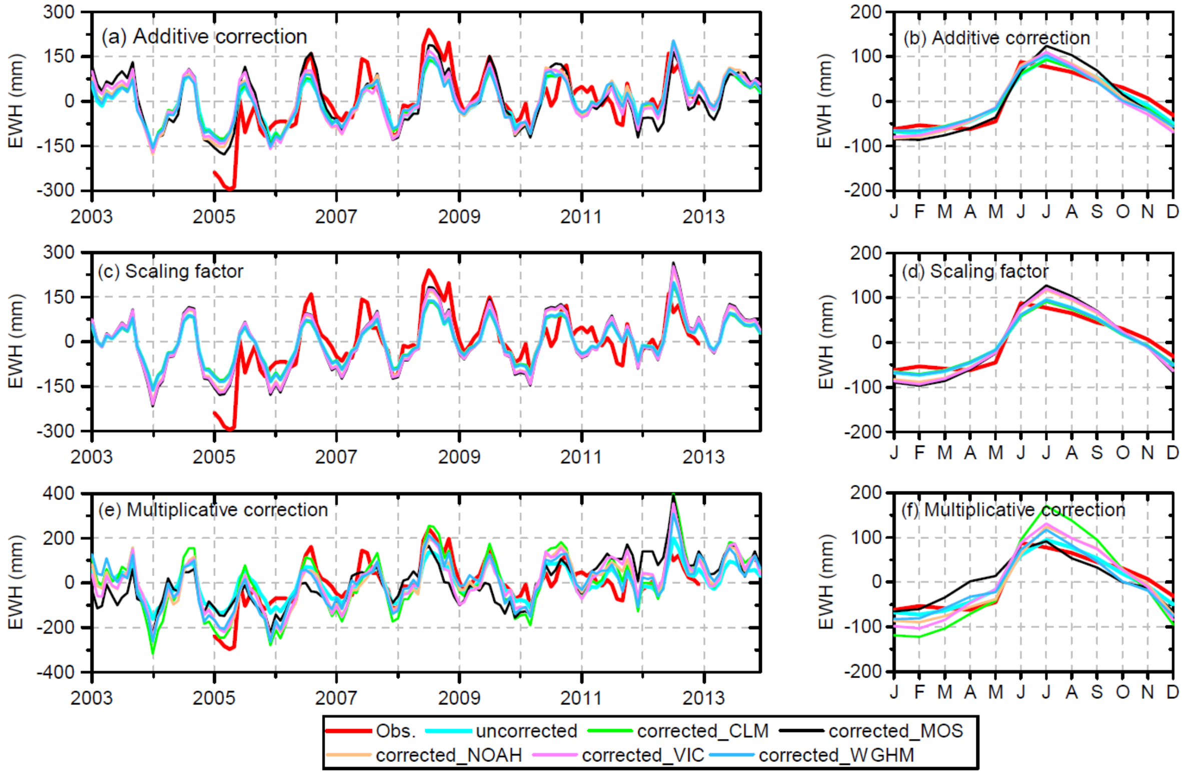

Figure S3. The same as Figure S2, but for Guangxi.
